# Biomedical Indicators of Patients with Non-Puerperal Mastitis: A Retrospective Study

**DOI:** 10.3390/nu14224816

**Published:** 2022-11-14

**Authors:** Liang Shi, Jing Wu, Yue Hu, Xu Zhang, Zhi Li, Pei-Wen Xi, Ji-Fu Wei, Qiang Ding

**Affiliations:** 1 Jiangsu Breast Disease Center, the First Affiliated Hospital with Nanjing Medical University, 300 Guangzhou Road, Nanjing 210029, China; 2 Department of Pathology, the First Affiliated Hospital with Nanjing Medical University, 300 Guangzhou Road, Nanjing 210029, China; 3 Department of Pharmacy, Jiangsu Cancer Hospital & Jiangsu Institute of Cancer Research & The Affiliated Cancer Hospital of Nanjing Medical University, Nanjing 210029, China

**Keywords:** non-puerperal mastitis, mammary duct ectasia, granulomatous lobular mastitis, biomedical indicators, lipid metabolism

## Abstract

Background: Non-puerperal mastitis (NPM), a recurrent chronic inflammation of non-lactating breast, often proves tremendous difficulty in treatment, and it may give rise to its complicated symptoms and unclear etiology. Furthermore, the clinical morbidity rate of NPM has been increasing in recent years. Methods: Overall, 284 patients diagnosed with NPM were consecutively recruited as cases in this study, and patients with benign breast disease (n = 1128) were enrolled as control. The clinical, biomedical, and pathological indicators were analyzed. Univariate and multivariate logistic analysis were used to distinguish risks between NPM and benign breast mass patients. Furthermore, according to the pathological characteristics, the patients of NPM were classified into two subgroups: mammary duct ectasia (MDE) and granulomatous lobular mastitis (GLM). The differences of biomedical indicators between MDE and GLM groups were also analyzed. Results: Compared with benign breast mass group, the level of high-density lipoprotein (HDL-C) significantly decreased, while lipoprotein(a) (Lp(a)) and blood glucose (GLU) both increased in NPM group. According to univariate and multivariate logistic analysis, the onset age and HDL-C were generally decreased, while Lp(a) and GLU were increased in NPM group. The onset age, HDL-C, Lp(a), and GLU were modeled to distinguish NPM and benign breast mass. Significant differences were also observed between MDE and GLM patients in biomedical indicators, such as lipoprotein(a) (Lp(a)), lactate dehydrogenase (LDH), creatine kinase (CK), total cholesterol (TC), and so on. Conclusions: Our results indicated for the first time that biomarkers were associated with NPM. The biomedical indicators involved in lipid metabolism might be important factors in the development and treatment of NPM. In addition, MDE and GLM are two diseases with different inflammatory states of NPM. These findings would be helpful for a better understanding of NPM and give us some insights to develop new diagnostic and therapeutic strategies.

## 1. Introduction

Mastitis is a common type of benign breast lesion with a diverse array of clinical presentations. It is an inflammatory condition of the breast, of which symptoms include sudden onset of pain and tenderness, swelling, erythema, warmth, and fever [1]. Inflammatory diseases are rare during the non-lactating phase but lead to considerable morbidity and difficulty in diagnosis [2]. Non-puerperal mastitis (NPM), also known as non-lactating mastitis, encompasses all the causes of inflammatory changes in the female breast and mammilla not related to lactation [3]. NPM is commonly sub-areolar and was first described as fistulas of lactiferous ducts by Zuska et al. [4]. Breast abscesses, as a complication, develop in 3–11% of women with mastitis, approximately 90% of which are sub-areolar. Furthermore, mammary fistulae, the complication of breast infectious, occurs in 1–2% of women with mastitis [5]. Non-infectious NPM is generally subclassified as mammary duct ectasia (MDE) and granulomatous lobular mastitis (GLM), while infectious NPM most commonly describes an infectious abscess (IAB) [6].

Mammary duct ectasia (MDE), also named periductal mastitis or plasma cell mastitis (PCM), is presented with nipple discharge and nipple retraction, which mimics malignancy [7]. Interestingly, at the histopathological level, it was primarily defined as a chronic inflammation of the breast with dilation of the mammary duct, plasma cell infiltration, and abscess formation [8,9]. In recent years, the morbidity rate of MDE has risen rapidly. Despite this, the clinical presentations of MDE are not specific, and the etiology of MDE is still unclear. Several factors have been reported as being associated with increased risks for MDE, such as smoking, obesity, and diabetes mellitus [8,10]. Recent findings have indicated that Interleukin-6 (IL-6)/Signal Transducer and Activator of Transcription 3 (STAT3) pathway, which was vital for the development and survival of plasma cells, also played an important role in the development of PCM [11]. Nevertheless, all of these risks were identified based on foreign small case reports and lack of systematic epidemiological cases [10]. To date, few studies have been reported on the correlation between the factors, such as lipid metabolism and immune factors of MDE.

Idiopathic granulomatous mastitis (IGM) is a rare, chronic, non-malignant, and non-life-threatening breast disease. Although it is a benign inflammatory disease of breast, it clinically and mammographically mimics breast cancer [12,13]. A palpable mass in the breast is the most common complaint, but nipple retraction, hyperemia in breast skin, oedema, ulceration, and fistula development during the chronic period are also potential complaints. However, the etiology of IGM remains elusive. Various factors have been indicated to play a role in disease etiology, including hormonal imbalance, autoimmunity, unknown microbiological agents, smoking, and α1-antitrypsin deficiency [14]. Diagnosis of IGM can be difficult and frequently delayed because it is diagnosed by the identification of granulomatous inflammation of the biopsied breast tissues without evidence of *Mycobacterium tuberculosis* infection [15]. Once diagnosed, treatment is often difficult and prolonged [16], and its treatment remains controversial. The proper management aims to achieve a good cosmetic effect and low recurrence rate by the integration of medical and surgical treatment. In some literatures, complete surgical excision, with or without corticosteroids and immunosuppression, remains controversial [17,18]. Although IGM is a benign breast disease, repeated surgical interventions may lead to poor cosmetic results.

Opinions regarding the etiology and mechanism of NPM vary, and there are also controversies about the optimal treatment. There are very few well-designed investigations tending to explore the risk factors and possible causes of NPM in Chinese patients. In this study, besides exploring the possible etiologic risk factors for NPM, the data were also used to compare the differences of clinical characteristics and biochemical results between NPM and benign breast mass patients. Furthermore, the data were analyzed for the differences between MDE and GLM subgroups in clinical, imaging, and pathological manifestations as well as treatment. Interestingly, the results showed that NPM was closely correlated to some lipid metabolism indicators in Chinese patients, which may provide important clues for the diagnosis and treatment of NPM in the future. Whether or not immune response plays an important role in the occurrence and development of mastitis remains to be addressed in future research.

## 2. Materials and Methods

### 2.1. Patients and Data Collection

This retrospective study was reviewed and approved by the Ethics Committee of Nanjing Medical University (approval ID: (2022)931). All patients involved in this study signed dedicated written informed consents.

The retrospective analysis was carried out in the First Affiliated Hospital with Nanjing Medical University. The patients enrolled in this study underwent treatment from June 2009 to April 2018. The following data were collected: age, menarche age, menstrual age, kinds of disease, onset time, and other characteristics, which were collected at the time of admission. In addition, the following information was also obtained: clinical, ultrasound, pathological features, and treatments of NPM. Otherwise, the corresponding information of the patients with benign breast mass was also detected. More importantly, another 188 patients were diagnosed with NPM by pathological evaluation after needle aspiration or operation.

### 2.2. Pathological Diagnosis

All the pathological features of NPM were randomly reviewed double-blinded by two pathologists, and other possible causes of mammary inflammation, such as breast tuberculosis, fat necrosis, as well as inflammatory due to lactation or pregnancy, were also ruled out.

### 2.3. Statistical Analysis

All statistical analyses were performed by IBM SPSS Statistics Version 20.0 (IBM Corp., Armonk, NY, USA). The mean values of quantitative datum were expressed as mean ± standard deviation (SD) on the condition of normal distribution. Chi-square test or Fisher’s exact test was applied in categorical variables, while two-sample Student’s *t*-test was applied to compare the difference between the means of continuous variables. The rank-sum test was used to estimate ordinal categorical data. The comparisons of biomedical indicators were made with rank-sum test, and the results were presented with median (M) and the interquartile range (Q1, Q3). The potential associated with identifying the risk factors between NPM and benign breast mass was investigated by using univariate and multivariate logistic regression analyses. Further, odds ratio (OR) with 95% confidence interval (95% CI) were used to differentiate NPM and benign breast mass. The statistical analyses of pathological characteristics were also presented between MDE and GLM. The statistically significant difference was defined as *p* ≤ 0.05.

## 3. Results

### 3.1. Baseline Characteristics and Biomedical Indicators of NPM and Benign Breast Mass Patients

We retrospectively reviewed 284 hospitalized patients with NPM and 1128 patients with benign breast mass, respectively. The clinical characteristics of all subjects were shown in Table S1. Compared with benign breast mass group, younger age (35.6 ± 9.8 years) and menarche age (14.3 ± 1.5 years) were reported in the NPM group. In addition, more patients with childbearing history (250/270, 92.59%), breastfeeding history (67/83, 80.72%), and high blood pressure (17/284, 5.99%) were reported in NPM group. However, relatively more patients (139/1122, 12.39%) with diabetes were found in the benign breast mass group. The longest breastfeeding median time of these two groups were 10 months.

Significant differences in biomedical indicators were discovered via comparing the basic information of NPM with benign breast mass patients. It was showed that high-density lipoprotein cholesterol (HDL-C) (median 1.25 mmol/L), urea (median 4.0 mmol/L), and creatinine (Cr) (median 50.4 μmol/L) were decreased in NPM group compared with benign breast mass group. Otherwise, it was observed that lipoprotein(a) (Lp(a)) (median 159 mg/L) and blood glucose (GLU) (median 5.18 mmol/L) were increased in the NPM group. There were no significant differences in alanine aminotransferase (ALT), aspartate aminotransferase (AST), glutamyl transpeptidase (GGT), lactate dehydrogenase (LDH), creatine kinase (CK), total cholesterol (TC), triglyceride (TG), and low-density lipoprotein cholesterol (LDL-C) between the NPM group and benign breast mass group. All the results were depicted in Table 1.

### 3.2. Baseline Characteristics, Clinical and Ultrasound Features, and Treatment of Non-Puerperal Mastitis Patients

To further describe the clinical features of NPM, another 188 pathological sections were examined again. Among the 188 NPM patients, 150 patients were diagnosed with MDE, and 38 patients were diagnosed with GLM. All the basic information was demonstrated in Table S2. According to the gathered information, we discovered that patients in the MDE group had a higher rate of congenital nipple retraction. However, the recurrence risks and other indicators were similar between MDE and GLM. In addition, the remaining important clinical findings, namely skin ulceration, suppuration, mass texture, and mobility, were also found in MDE and GLM cohorts and depicted in Table S3. In the meantime, there was no significant difference between MDE and GLM groups when comparing their ultrasound features and treatment (Table S3).

### 3.3. Univariate and Multivariate Logistic Analysis for Risks between Non-Puerperal Mastitis and Benign Breast Mass 

To examine the potential risk factors between NPM and benign breast mass patients, a univariate logistic analysis was performed. All the results were shown in Table 2. An analysis of the full dataset indicated that, in the NPM group, the onset age was generally lower (OR, 1.073; 95% CI, 1.059–1.008; *p* < 0.001), and the incidence of hypertension was slightly higher (OR, 2.221; 95% CI, 1.318–3.741; *p* = 0.003). Furthermore, the levels of Lp(a) (OR, 0.999; 95% CI, 0.998–1.000; *p* = 0.022) and GLU (OR, 0.845; 95% CI, 0.732–0.977; *p* = 0.023) were higher in NPM group, while the levels of HDL-C (OR, 4.514; 95% CI, 1.735–11.746; *p* = 0.002), urea (OR, 1.375; 95% CI, 1.081–1.748; *p* = 0.009), and Cr (OR, 1.051; 95% CI, 1.091–1.084; *p* = 0.002) were lower in the NPM group. All the factors in the univariate analysis with *p*-values ≤ 0.05 were regarded as candidate predictors for a multivariate logistic regression model.

**Figure 1:**
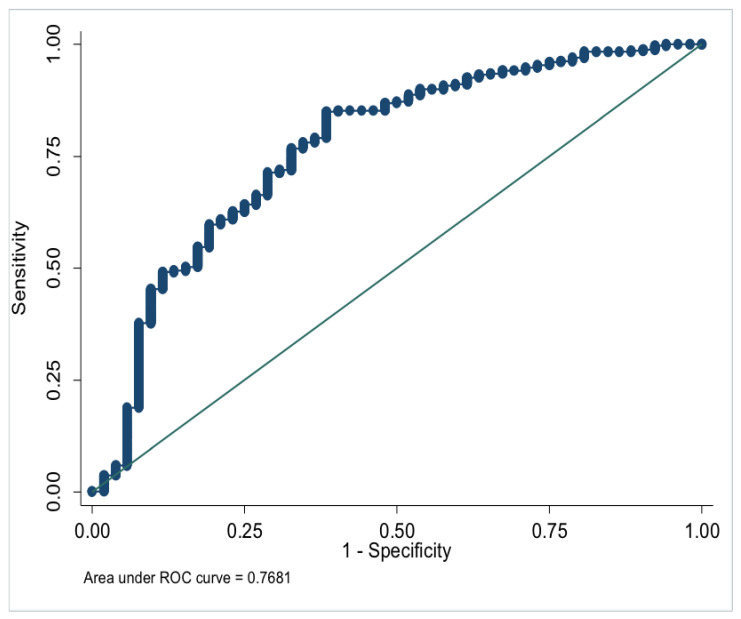
ROC of model parameters to distinguish NPM and benign breast mass.

In the multivariate analysis, the onset age (OR, 1.065; 95% CI, 1.021–1.110; *p* = 0.003) and HDL-C (OR, 4.386; 95% CI, 1.206–15.946; *p* = 0.025) were generally lower in the NPM group. Lp(a) (OR, 0.998; 95% CI, 0.997–1.000; *p* = 0.021) and GLU (OR, 0.567; 95% CI, 0.373–0.862; *p* = 0.008) were higher in NPM group (Table 3). Meanwhile, the onset age, HDL-C, Lp(a), and GLU were modeled to distinguish NPM and benign breast mass, with AUC = 0.768 (Figure 1).

### 3.4. Biomedical Indicators of MDE and GLM Patients

Overwhelming differences (*p* ≤ 0.05) were pointed out in biomedical indicators between MDE and GLM groups. All the results were depicted in Table 4. It was found that LDH (median 174.5 U/L), CK (median 77.0 U/L), TC (median 4.7 mmol/L), and HDL-C (median 1.36 mmol/L) were significantly increased in MDE group than those in GLM group. However, the expression of LDL-C (median 0.93 mmol/L) was remarkably decreased in MDE group compared with GLM group. Other indicators, such as AST, ALP, GGT, TG, Lp(a), GLU, urea, and Cr, were also depicted in Table 4, while there were no differences in these indicators between MDE and GLM patients. 

### 3.5. Pathological Characteristics of Non-Puerperal Mastitis

**Figure 2:**
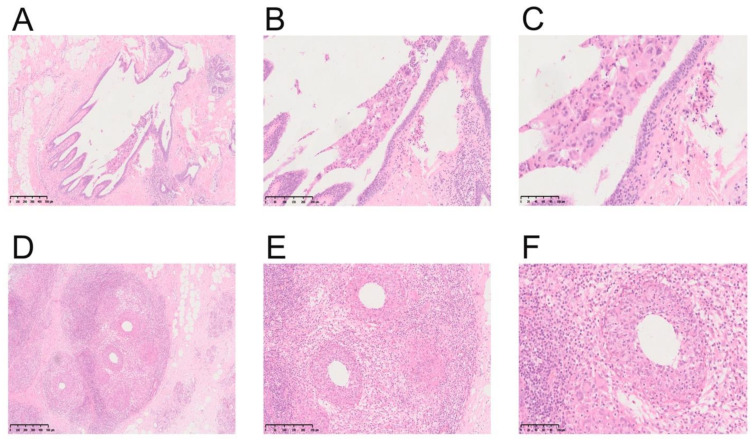
Representative illustrations of MDE and GLM. (**A**) Low-power magnification of MDE (HE, ×40). (**B**) Medium-power magnification of MDE (HE, ×100). (**C**) High-power magnification of MDE (HE, ×200). (**D**) Low-power magnification of GLM (HE, ×40). (**E**) Medium-power magnification of GLM (HE, ×100). (**F**) High-power magnification of GLM (HE, ×200).

By analyzing the pathological features of MDE and GLM, we found that the characteristic structure of MDE group was ductal expansion (46/150, 30.67%), while GLM was dominated with lobular structure inflammation (17/38, 44.74%). Moreover, it was shown by careful observation under a high-power microscope that large amounts of plasma cells (146/150, 97.33%), lymphocytes (137/150, 91.33%), and neutrophils (122/150, 81.33%) were infiltrated in the MDE group. However, plasma cell (24/38, 63.16%), lymphocyte (23/38, 60.53%), and neutrophil (21/38, 55.26%) infiltrations were also uncovered in the GLM group, and the proportion of these cells was significantly lower than that in the MDE group. In addition, histiocytic responses were also found in MDE and GLM, accounting for 44.00% (66/150) and 13.16% (5/38), respectively. Granulomatous inflammation was mainly presented in GLM (32/38, 84.21%), and a smaller proportion (74/150, 49.33%) was found in MDE. All the indicators were demonstrated in Table 5. To explore the typical microscopic characteristics of the NPM group, the pathological characteristics were observed under low-, medium-, and high-power microscopes. In the MDE group, the significantly dilated duct was illustrated in low power (40 times amplification), while in medium power (100 times amplification), a large number of lymphocytes and plasma cells were infiltrated in the mammary stroma, and in high power (200 times amplification), exfoliated cells and lipid debris were accumulated in the dilated duct. However, in GLM group, the lesions extended to the terminal ductal lobular structure of mammary gland were observed in low power (40 times amplification). In medium power (100 times amplification), plenty of lymphocytes infiltrated the mammary glands, and polynuclear cell reaction and lipid absorption vacuoles were seen under high power (200 times amplification). The typical histopathological figures of MDE and GLM were presented in Figure 2.

## 4. Discussions

Non-puerperal mastitis (NPM) is a relatively uncommon benign breast entity and accounts for about 4–5% of all benign breast lesions [4,6,19]. As a main source of long-term breast diseases, it is also a chronic inflammation in females unrelated to pregnancy and breastfeeding, which can be difficult to treat [20]. However, in recent years, the incidence and recurrence of NPM have risen rapidly. Thus far, the etiology of NPM is still unknown.

In this paper, we sought to identify the risk factors of NPM in China by comparing it with benign breast mass. Based on the presentation, only a small percentage of patients were found to have high blood pressure and diabetes, which was significantly different from previous studies. The development of mastitis may be related to race and living environment. All of the stated risks were identified based on small-scale projects abroad, and thus, there is a lack of systematic epidemiological cases [10]. Surprisingly, the biomedical results of NPM and benign breast mass patients further showed that the expression of lipoprotein(a) (Lp(a)) increased, and high-density lipoprotein (HDL-C) decreased in NPM group. As we all know, Lp(a) is an independent cardiovascular risk factor, and it is also more strongly associated with cardiovascular and all-cause mortality than low-density lipoprotein [21,22,23]. Additionally, contemporary general population study has indicated that high plasma levels of Lp(a) were also associated with increased risk of ischemic stroke [24]. It has been shown that plasma HDL-C concentrations were inversely associated with the risk of cardiovascular events [25,26]. HDL-C possesses anti-inflammatory properties and regulates both innate and adaptive immune response, and it plays an important role in reducing atherosclerosis and inflammation [27]. However, the relationships between Lp(a), HDL-C, and NPM are unclear, which shall provide some clues for our further study. Furthermore, the baseline characteristics of NPM based on MDE and GLM classifications showed that patients in the MDE group had a higher rate of congenital nipple retraction. Meanwhile, in previous foreign studies, smoking, obesity, and diabetes mellitus were considered as the major risk factors of NPM [10,28]. This disease typically presents with breast mass, nipple retraction, sinus formation, and axillary lymphadenopathy [29]. Consistent with previous reports, all the patients had the above clinical manifestations. Notably, the younger the patients, the less likely they suffered from diabetes mellitus and smoking, which may be the major differences between domestic and foreign studies.

Mammary duct ectasia (MDE) is histopathologically defined as a chronic inflammation of the breast with dilation of the mammary duct, plasma cell infiltration, and abscess formation [30,31]. Nevertheless, the clinical presentations of MDE are not specific, which are easily confused with breast cancer in clinical and imaging manifestations [32,33]. In previous studies, smoking, obesity, and diabetes mellitus were considered as the risk factors of MDE. To some extent, bacterial infection was thought to be a possible etiological factor of MDE [8], and it was also discovered that IL-6/JAK2/STAT3 signaling activity was significantly elevated in the development of plasma cell mastitis via constructing mouse models [34]. All of this evidence implied that the development of PCM might be associated with immunity, which would provide clues for our follow-up research.

Idiopathic granulomatous mastitis (IGM), described as a rare chronic benign inflammatory disorder of the breast, was first defined by Kessler and Wolloch in 1972 [35]. The locally typical clinical manifestations of IGM comprise breast mass, skin ulceration with draining sinus tracts, and a raised skin lesion with associated palpable masses; some patients develop limb erythema nodosum [13,16]. Nevertheless, the etiology of the GLM remains exclusive. Several factors were found to be related to GLM, such as microbiological agents, hormonal effects, and immunologic disorders [14]. Additionally, the treatment of GLM remains controversial. The main treatment approaches include antibiotics with repeated drainage, wide surgical excision or mastectomy, oral steroids, immunosuppression with methotrexate, and close follow-up. There are on-going debates regarding which is the most appropriate treatment [36]. The recurrence rate remains high even after mastectomy; therefore, nonsurgical treatment is becoming more popular. To some extent, empirical treatment might play a role in the process of treatment [37].

Due to the differences between MDE and GLM, we divided NPM into MDE and GLM subgroups according to pathological results. On one hand, we analyzed the difference of demographic characteristics between MDE and GLM. We systematically analyzed the differences between MDE and GLM in clinical and ultrasound features, treatment, and biomedical indicators. We found similar clinical manifestations between MDE and GLM, which indicated that it was difficult to distinguish MDE and GLM. The above analysis showed that MDE and GLM behaved similarly whether they were different states of the same disease or even actually different diseases. Considering that pathological diagnosis is the cornerstone of any diagnosis and the gold standard of differential diagnosis, we analyzed the pathological results of MDE and GLM to solve the puzzle. Large amounts of plasma cells, lymphocytes, and neutrophiles were infiltrated in MDE, which suggested that MDE might be associated with acute inflammation. Although part of the above cells could also be observed in GLM, typical granulomatous inflammation was mainly presented in GLM. Additionally, pathological manifestations might be one of the points of differentiation between MDE and GLM. Furthermore, we also analyzed the biomedical indicators of MDE and GLM patients to identify them comprehensively. To our surprise, compared to the GLM group, LDH, CK, TC, and HDL-C were highly expressed, whereas LDL-C was remarkably decreased in MDE. Thus far, no research in these areas has explicitly focused on the biomedical indicators, and people know little about the occurrence and development of NPM’s link to lipid metabolism; this paper aims to help fill this research lacuna.

There are several limitations in the present study. For example, only sonography examination was contained in the analysis of imaging results. Nevertheless, the manifestations of NPM were prone to be confused with breast cancer. Meanwhile, the overall smoking rate of Chinese women is low. In recent years, China has strengthened the prohibition of smoking in indoor public places and workplaces to avoid the harm caused by second-hand smoke. Therefore, there were no smokers in our study. Given this study was just a retrospective research, the available image data and clinical variables were limited. As the number of cases is relatively small, and the incidence of NPM is relatively low, the research in our paper could represent part of the patients. In the future study, we will improve imagological examination, for example, with magnetic resonance and mammography. Moreover, we shall conduct our research involving more NPM cases and comprehensive information, such as obesity, smoking, alcohol, physical activity, and so on.

In summary, non-puerperal mastitis (NPM) describes a group of chronic inflammatory conditions of the non-lactating breast. This retrospectively analysis highlights potential etiological factors on the onset of NPM but does not address the primary etiological factors related to the development of NPM. The lipid metabolism is believed to perhaps be a significant factor of NPM, and it still needs to be a focus, and new ideas should be provided for future diagnosis and treatment. Although we have discovered the differences in biomedical indicators and pathological characteristics, we still need to further study the causes and mechanisms of these differences.

## 5. Conclusions

In this retrospective study comparing with benign breast mass patients, we observed that the onset age and menarche age were younger in NPM patients. Biomedical indicators, such as high-density lipoprotein cholesterol (HDL-C), urea, and creatinine (Cr), were lower-expressed in the NPM group. Meanwhile, lipoprotein(a) (Lp(a)) and blood glucose (GLU) increased in the NPM group. The biomedical indicators involved in lipid metabolism might be important factors in the development and treatment of NPM. In the multivariate analysis, the onset age, HDL-C, Lp(a), and GLU were modeled to distinguish NPM and benign breast mass. Moreover, MDE and GLM are two diseases with different inflammatory states of NPM. These findings would be helpful for a better understanding of NPM and give us some insights to develop new diagnostic and therapeutic strategies. Due to the limitation of this retrospective study, we will conduct comprehensive research on the mechanisms of biomedical indicators in NPM in the future.

## Figures and Tables

**Table 1:** The biomedical indicators of NPM and benign breast mass patients.

Variable	Non-Puerperal Mastitis (284)	Benign Breast Mass (1128)	*p*-Value
ALT (U/L) M (25%, 75%)	13.7 (11.8, 18.8)	15.6 (12.0, 21.4)	0.366
AST (U/L) M (25%, 75%)	18.9 (15.1, 24.0)	19.6 (16.2, 23.6)	0.36
GGT (U/L) M (25%, 75%)	19.8 (15.0, 25.1)	14.9 (11.3, 21.6)	0.455
LDH (U/L) M (25%, 75%)	174 (158, 198)	168 (149,189)	0.156
CK (U/L) M (25%, 75%)	62.6 (52.0, 80.0)	69.0 (53.6, 91.2)	0.355
TC (mmol/L) M (25%, 75%)	4.5 (4.1, 4.9)	4.46 (3.85, 5.07)	0.964
TG (mmol/L) M (25%, 75%)	1.15 (0.78, 1.62)	0.94 (0.70, 1.35)	0.081
HDL-C (mmol/L) M (25%, 75%)	1.25 (1.08, 1.44)	1.4 (1.2, 1.6)	0.002
LDL-C (mmol/L) M (25%, 75%)	2.81 (2.50, 3.20)	2.65 (2.18, 3.21)	0.287
Lp(a) (mg/L) M (25%, 75%)	159 (59, 398)	125 (59, 246)	0.022
GLU (mmol/L) M (25%, 75%)	5.18 (4.85, 5.46)	4.93 (4.60, 5.39)	0.023
Urea (mmol/L) M (25%, 75%)	4.0 (3.6, 4.9)	4.6 (3.8, 5.5)	0.009
Cr (μmol/L) M (25%, 75%)	50.4 (46.3, 57.1)	55.0 (46.0, 62.0)	0.002

**Table 2:** Univariate logistic regression analysis of selected risk factors between non-puerperal mastitis and benign breast mass.

Variable	Non-Puerperal Mastitis (284)	Benign Breast Mass (1128)	OR	*p*-Value	95% CI
Age (years)	35.6 ± 9.8	44.3 ± 12.3	1.073	<0.001	1.059–1.088
High blood pressure			2.221	0.003	1.318–3.741
Yes	17	39			
No	267	1082			
AST (U/L) M (25%, 75%)	18.9 (15.1, 24.0)	19.6 (16.2, 23.6)	0.991	0.36	0.971–1.011
GGT (U/L) M (25%, 75%)	19.8 (15.0, 25.1)	14.9 (11.3, 21.6)	0.999	0.455	0.996–1.002
LDH (U/L) M (25%, 75%)	174 (158, 198)	168 (149, 189)	0.997	0.156	0.992–1.001
CK (U/L) M (25%, 75%)	62.6 (52, 80)	69 (53.6, 91.2)	1.004	0.355	0.996–1.012
TC (mmol/L) M (25%, 75%)	4.5 (4.1, 4.9)	4.46 (3.85, 5.07)	1.007	0.964	0.754–1.343
TG (mmol/L) M (25%, 75%)	1.15 (0.78, 1.62)	0.94 (0.70, 1.35)	0.761	0.081	0.560–1.034
HDL-C (mmol/L) (25%, 75%)	1.25 (1.08, 1.44)	1.4 (1.2, 1.6)	4.514	0.002	1.735–11.746
LDL-C (mmol/L) M (25%, 75%)	2.81 (2.5, 3.2)	2.65 (2.18, 3.21)	0.837	0.287	0.603–1.161
Lp(a) (mg/L) M (25%, 75%)	159 (59, 398)	125 (59, 246)	0.999	0.022	0.998–1.000
GLU (mmol/L) M (25%, 75%)	5.18 (4.85, 5.46)	4.93 (4.6, 5.39)	0.845	0.023	0.732–0.977
Urea (mmol/L) M (25%, 75%)	4.0 (3.6, 4.9)	4.6 (3.8, 5.5)	1.375	0.009	1.081–1.748
Cr (μmol/L) M (25%, 75%)	50.4 (46.3, 57.1)	55 (46, 62)	1.051	0.002	1.019–1.084

**Table 3:** Multivariate logistic regression analysis of selected risk factors between non-puerperal mastitis and benign breast mass.

Variable	Non-Puerperal Mastitis (284)	Benign Breast Mass (1128)	OR	*p*-Value	95% CI
Age (years)	35.6 ± 9.8	44.3 ± 12.3	1.065	0.003	1.021–1.110
High blood pressure			6.036	0.108	0.674–54.081
Yes	17	39			
No	267	1082			
HDL-C (mmol/L) M (25%, 75%)	1.25 (1.08, 1.44)	1.4 (1.2, 1.6)	4.386	0.025	1.206–15.946
Lp(a) (mg/L) M (25%, 75%)	159 (59, 398)	125 (59, 246)	0.998	0.021	0.997–1.000
GLU (mmol/L) M (25%, 75%)	5.18 (4.85, 5.46)	4.93 (4.6, 5.39)	0.567	0.008	0.373–0.862
Urea (mmol/L) M (25%, 75%)	4.0 (3.6, 4.9)	4.6 (3.8, 5.5)	1.159	0.37	0.840–1.600
Cr (μmol/L) M (25%, 75%)	50.4 (46.3, 57.1)	55 (46, 62)	1.037	0.081	0.995–1.080

**Table 4:** Biomedical indicators of non-puerperal mastitis patients.

Variable	Mammary Duct Ectasia (150)	Granulomatous Mastitis (38)	*p*-Value
AST (U/L) M (25%, 75%)	18.5 (14.8, 23.4)	19.8 (15.6, 48.9)	0.5177
ALP (U/L) M (25%, 75%)	74.3 (59.4, 91.0)	68.0 (57.0, 88.0)	0.6894
GGT (U/L) M (25%, 75%)	19.7 (14.1, 27.7)	17.7 (14.1, 28.8)	0.9848
LDH (U/L) M (25%, 75%)	174.5 (159.5, 204.5)	156.5 (141.0, 168.0)	0.03
CK (U/L) M (25%, 75%)	77.0 (52.5, 87.9)	53.5 (40.5, 63.5)	0.0346
TC (mmol/L) M (25%, 75%)	4.7 (4.5, 5.4)	4.1 (3.6, 4.5)	0.0102
TG (mmol/L) M (25%, 75%)	0.93 (0.68, 1.61)	1.30 (0.90, 1.77)	0.4242
HDL-C (mmol/L) M (25%, 75%)	1.36 (1.15, 1.64)	1.07 (1.04, 1.15)	0.0026
LDL-C (mmol/L) M (25%, 75%)	0.93 (2.62, 3.44)	2.64 (2.11, 2.91)	0.0355
Lp(a) (mg/L) M (25%, 75%)	246 (96, 456)	111 (48, 159)	0.0771
GLU (mmol/L) M (25%, 75%)	5.23 (4.87, 5.43)	4.92 (4.66, 5.66)	0.7533
Urea (mmol/L) M (25%, 75%)	4.0 (3.5, 4.9)	3.8 (3.4, 4.3)	0.4436
Cr (μmol/L) M (25%, 75%)	49.4 (46.2, 57.6)	43.6 (42.2, 50.4)	0.0648

**Table 5:** Pathological characteristics of non-puerperal mastitis.

Variable	Mammary Duct Ectasia (150)	Granulomatous Mastitis (38)	*p*-Value
Duct and lobular			<0.001
No	76	6	
Duct	46	4	
Lobular	7	17	
Duct and lobular	21	11	
Plasma cell			<0.001
Yes	146	24	
No	4	14	
Lymphocyte			
Yes	137	23	<0.001
No	13	15	
Neutrophil			
Yes	122	21	0.002
No	28	17	
Eosinophil			1.00
Yes	1	0	
No	149	38	
Histocyte response			<0.001
Yes	66	5	
No	84	33	
Multicenter giant cell response			0.579
Yes	60	13	
No	90	25	
Granulomatous inflammation			<0.001
Yes	74	32	
No	76	6	

## Data Availability

The data used and analyzed in this study are available from the corresponding authors on reasonable request. The data are not publicly available due to privacy issues.

## Supplementary Materials

The following supporting information can be downloaded at: https://www.mdpi.com/article/10.3390/nu14224816/s1. Table S1: Baseline characteristics between non-puerperal mastitis and benign breast mass patients; Table S2: Baseline characteristics of non-puerperal mastitis patients; Table S3: Clinical, ultrasound features and treatment of non-puerperal mastitis patients.

## References

[1] An J.K., Woo J.J., Lee S.A. (2016). Non-puerperal mastitis masking pre-existing breast malignancy: Importance of follow-up imaging. Ultrasonography.

[2] Nair C.G., Jacob P., Menon R.R. (2015). Inflammatory diseases of the non-lactating female breasts. Int. J. Surg..

[3] Tan H., Li R., Peng W., Liu H., Gu Y., Shen X. (2013). Radiological and clinical features of adult non-puerperal mastitis. Br. J. Radiol..

[4] Zuska J.J., Crile G., Ayres W.W. (1951). Fistulas of lactifierous ducts. Am. J. Surg..

[5] Boakes E., Woods A., Johnson N., Kadoglou N. (2018). Breast Infection: A Review of Diagnosis and Management Practices. Eur. J. Breast Health.

[6] Kasales C.J., Han B., Smith J.S., Chetlen A.L., Kaneda H.J., Shereef S. (2014). Nonpuerperal mastitis and subareolar abscess of the breast. AJR Am. J. Roentgenol..

[7] Leong P.W., Chotai N.C., Kulkarni S. (2018). Imaging Features of Inflammatory Breast Disorders: A Pictorial Essay. Korean J. Radiol..

[8] Liu L., Zhou F., Wang P., Yu L., Ma Z., Li Y., Gao D., Zhang Q., Li L., Yu Z. (2017). Periductal Mastitis: An Inflammatory Disease Related to Bacterial Infection and Consequent Immune Responses?. Mediat. Inflamm..

[9] Zhang Y., Zhou Y., Mao F., Guan J., Sun Q. (2018). Clinical characteristics, classification and surgical treatment of periductal mastitis. J. Thorac. Dis..

[10] Gollapalli V., Liao J., Dudakovic A., Sugg S.L., Scott-Conner C.E., Weigel R.J. (2010). Risk factors for development and recurrence of primary breast abscesses. J. Am. Coll. Surg..

[11] Liu Y., Zhang J., Zhou Y.H., Jiang Y.N., Zhang W., Tang X.J., Ren Y., Han S.P., Liu P.J., Xu J. (2015). IL-6/STAT3 signaling pathway is activated in plasma cell mastitis. Int. J. Clin. Exp. Pathol..

[12] Calis H., Kilitci A. (2018). Granulomatous Mastitis Concurrence with Breast Cancer. Eur. J. Breast Health.

[13] Mahmodlou R., Dadkhah N., Abbasi F., Nasiri J., Valizadeh R. (2017). Idiopathic granulomatous mastitis: Dilemmas in diagnosis and treatment. Electron. Physician.

[14] Altintoprak F., Kivilcim T., Ozkan O.V. (2014). Aetiology of idiopathic granulomatous mastitis. World J. Clin. Cases.

[15] Co M., Cheng V.C.C., Wei J., Wong S.C.Y., Chan S.M.S., Shek T., Kwong A. (2018). Idiopathic granulomatous mastitis: A 10-year study from a multicentre clinical database. Pathology.

[16] Fazzio R.T., Shah S.S., Sandhu N.P., Glazebrook K.N. (2016). Idiopathic granulomatous mastitis: Imaging update and review. Insights Imaging.

[17] Gurleyik G., Aktekin A., Aker F., Karagulle H., Saglamc A. (2012). Medical and surgical treatment of idiopathic granulomatous lobular mastitis: A benign inflammatory disease mimicking invasive carcinoma. J. Breast Cancer.

[18] Kok K.Y., Telisinghe P.U. (2010). Granulomatous mastitis: Presentation, treatment and outcome in 43 patients. Surgeon.

[19] Zhang L., Hu J., Guys N., Meng J., Chu J., Zhang W., Liu A., Wang S., Song Q. (2018). Diffusion-weighted imaging in relation to morphology on dynamic contrast enhancement MRI: The diagnostic value of characterizing non-puerperal mastitis. Eur. Radiol..

[20] Yanai A., Hirabayashi S., Ueda K., Okabe K. (1987). Treatment of recurrent subareolar abscess. Ann. Plast. Surg..

[21] Langsted A., Kamstrup P.R., Nordestgaard B.G. (2019). High lipoprotein(a) and high risk of mortality. Eur. Heart J..

[22] Klingel R., Heibges A., Fassbender C. (2019). Lipoprotein(a) and mortality-a high risk relationship. Clin. Res. Cardiol. Suppl..

[23] Brandao J.A.M., Meireles-Brandao L.R., Coelho R., Rocha-Goncalves F. (2019). Lipoprotein(a) as a key target in combined therapeutic approaches for cardiovascular disease. Rev. Port. Cardiol..

[24] Langsted A., Nordestgaard B.G., Kamstrup P.R. (2019). Elevated Lipoprotein(a) and Risk of Ischemic Stroke. J. Am. Coll. Cardiol..

[25] Rye K.A. (2013). High density lipoprotein structure, function, and metabolism: A new Thematic Series. J. Lipid Res..

[26] Jia C., Anderson J.L.C., Gruppen E.G., Lei Y., Bakker S.J.L., Dullaart R.P.F., Tietge U.J.F. (2021). High-Density Lipoprotein Anti-Inflammatory Capacity and Incident Cardiovascular Events. Circulation.

[27] Hu J., Xi D., Zhao J., Luo T., Liu J., Lu H., Li M., Xiong H., Guo Z. (2016). High-density Lipoprotein and Inflammation and Its Significance to Atherosclerosis. Am. J. Med. Sci..

[28] Risager R., Bentzon N. (2010). [Smoking and increased risk of mastitis]. Ugeskr Laeger.

[29] Oltean H.N., Soliman A.S., Omar O.S., Youssef T.F., Karkouri M., Abdel-Aziz A., Hablas A., Blachley T., Tahri A., Merajver S.D. (2013). Risk factors for chronic mastitis in morocco and egypt. Int. J. Inflam..

[30] Ramalingam K., Srivastava A., Vuthaluru S., Dhar A., Chaudhry R. (2015). Duct Ectasia and Periductal Mastitis in Indian Women. Indian J. Surg..

[31] Dixon J.M. (1989). Periductal mastitis/duct ectasia. World J. Surg..

[32] Kim B.S., Lee J.H., Kim W.J., Kim D.C., Shin S., Kwon H.J., Park J.S., Park Y.M. (2013). Periductal mastitis mimicking breast cancer in a male breast. Clin. Imaging.

[33] Song L., Li L., Liu B., Yu D., Sun F., Guo M., Ruan Z., Zhang F. (2018). Diagnostic evaluations of ultrasound and magnetic resonance imaging in mammary duct ectasia and breast cancer. Oncol. Lett..

[34] Liu Y., Zhang J., Zhou Y.H., Zhang H.M., Wang K., Ren Y., Jiang Y.N., Han S.P., He J.J., Tang X.J. (2018). Activation of the IL-6/JAK2/STAT3 pathway induces plasma cell mastitis in mice. Cytokine.

[35] Kessler E., Wolloch Y. (1972). Granulomatous mastitis: A lesion clinically simulating carcinoma. Am. J. Clin. Pathol..

[36] Shin Y.D., Park S.S., Song Y.J., Son S.M., Choi Y.J. (2017). Is surgical excision necessary for the treatment of Granulomatous lobular mastitis?. BMC Womens Health.

[37] Liu L., Zhou F., Zhang X., Liu S., Liu L., Xiang Y., Guo M., Yu L., Wang F., Ma Z. (2018). Granulomatous Lobular Mastitis: Antituberculous Treatment and Outcome in 22 Patients. Breast Care.

