# Supplementary material for: Biomedical Indicators of Patients with Non-Puerperal Mastitis: A Retrospective Study
Source: Nutrients. 2022 Nov 14;14(22):4816. doi: 10.3390/nu14224816 (PMC9695051; doi:10.3390/nu14224816)
Supplement: Supplementary file 1 [file nutrients-14-04816-s001.zip › nutrients-1940221-supplementary.pdf]

Table S1. Baseline characteristics between non-puerperal mastitis and benign breast mass patients.

Variable	Non-puerperal mastitis (284)	Benign breast mass (1128)	P-value
Age (years)	35.6±9.8	44.3±12.3	<0.001
Menarche age (years) **	14.3±1.5	14.6±1.5	0.033
Childbearing history**			0.014
Yes	250 (92.59 %)	890 (87.17 %)	
No	20 (7.41 %)	131 (12.83 %)	
Breastfeeding history**			0.025
Yes	67 (80.72%)	590 (89.12 %)	
No	16 (19.28%)	72 (10.88 %)	
The longest breastfeeding time (months) ** M (25%, 75%)	10 (6,12)	10 (6,12)	0.959
High blood pressure **			0.054
Yes	17 (5.99 %)	39 (3.48 %)	
No	267 (94.01 %)	1082 (96.52%)	
Diabetes mellitus**			<0.001
Yes	2 (0.70%)	139 (12.39 %)	
No	282 (99.3%)	983 (87.61 %)	

** Some data was missing.

Table S2. Baseline characteristics of non-puerperal mastitis patients.

Variable	Mammary duct ectasia (150)	Granulomatous Mastitis (38)	P-value
Age (years)	36.2±9.8	33.4±7.4	0.1039
Menarche age (years) **	14.4±1.6	14.2±1.4	0.4364
Menstrual regularity**			0.091
Yes	125	33	
No	11	0	
Onset time (months) M (25%, 75%)	2.0 (0.7, 5.0)	2.0 (1.0, 3.0)	0.6595
Recurrence			0.896
Yes	25	6	
No	125	32	
Congenital nipple retraction**			0.034
Yes	14	1	
No	48	25	
Number of pregnancy ** M (25%, 75%)	2 (1, 2)	2 (1, 2)	0.9724
Number of breastfeeding ** M (25%, 75%)	1 (1, 1)	1 (1, 1)	0.3187
The longest breastfeeding time (months) ** M (25%, 75%)	11 (7, 12)	12 (6, 12)	0.9063

Breastfeeding of sick lateral breast**			0.442
Yes	29	9	
No	1	1	
Breast mass distribution			0.845
Left breast	69	20	
Right breast	75	17	
Bilateral breast	6	1	
Quadrant of mass			0.716
Nodular or no mass	5	2	
Outer upper quadrant	42	9	
Outer lower quadrant	18	8	
Inner lower quadrant	8	3	
Inner upper quadrant	25	4	
Areola area	26	6	
Multiple quadrants	25	6	
High blood pressure			0.362
Yes	8	0	
No	142	38	
Diabetes mellitus			1.00

Yes	1	0
No	149	38

** Some data was missing.

Table S3. Clinical, ultrasound features and treatment of non-puerperal mastitis patients.

Variable	Mammary duct ectasia (150)	Granulomatous Mastitis (38)	P-value
Epidermal redness**			0.261
Yes	44	10	
No	51	19	
Edema**			0.225
Yes	38	8	
No	54	20	
Skin temperature increasing**			0.336
Yes	14	2	
No	50	18	
Skin ulceration**			0.674
Yes	32	11	
No	63	18	
Suppuration**			0.074
Yes	25	14	
No	65	17	
Mass boundary**			
Clear	19	7	0.148
Unclear	91	16	
Mass texture**			0.67
Soft	47	8	
Hard	67	14	
Mobility**			0.132
Good	70	18	
Poor	37	4	

Duct expansion**			0.745
Yes	23	4	
No	90	19	
Glander structure disorder**			0.145
Yes	68	11	
No	38	12	
Axillary lymph node enlargement**			0.935
Yes	28	6	
No	76	17	
Antibiotic therapy**			0.088
Yes	76	21	
No	44	5	
Operative treatment**			
Partial mastectomy	138	33	0.256
Total mastectomy	11	5	

** Some data was missing.
